# Serum or Plasma Oncostatin M for Predicting Primary Non-Response to Tumor Necrosis Factor-α Antagonist Therapy in Inflammatory Bowel Disease: A Systematic Review and Meta-Analysis

**DOI:** 10.3390/clinpract16070124

**Published:** 2026-07-02

**Authors:** Maxwell A. Barffour, Elizabeth Karanja, Mustafa Gandhi, Susan S. Kais, Saurabh Kapur, Yezaz A. Ghouri

**Affiliations:** 1 Department of Medicine, Washington University School of Medicine, St. Louis, MO 63110, USA; 2 College of Medicine, The Ohio State University, Columbus, OH 43210, USA; elizabeth.karanja@osumc.edu; 3 Division of Gastroenterology and Hepatology, University of Missouri School of Medicine, Columbia, MO 65201, USA; mustafa.gandhi@health.missouri.edu; 4 Division of Gastroenterology and Hepatology, University of Cincinnati, Cincinnati, OH 45221, USA; kaisss@ucmail.uc.edu; 5 Division of Gastroenterology, Hepatology and Motility, University of Kansas School of Medicine, Kansas City, KS 64110, USA; skapur@kumc.edu; 6 Division of Gastroenterology, St. Louis University School of Medicine, St. Louis, MO 63110, USA; yezaz.ghouri@ssmhealth.com

**Keywords:** oncostatin M, Anti-TNF, inflammatory bowel disease, ulcerative colitis, Crohn’s disease

## Abstract

**Background**: Over 30% of patients with inflammatory bowel disease (IBD) experience primary non-response to tumor necrosis factor-α antagonists (anti-TNFs). Oncostatin M (OSM), a TNF-α-independent pro-inflammatory signaling cytokine, is emerging as a potential predictive biomarker of treatment response. Through a systematic review and meta-analysis, we assessed the utility of baseline serum or plasma OSM for predicting anti-TNF response in IBD patients. **Methods**: We searched PubMed, Embase, Cochrane Library, and Web of Science through March 2026 for studies reporting associations between baseline serum/plasma OSM and endoscopic or clinical response to anti-TNF therapy in adult IBD patients. We used a bivariate random-effects model to estimate pooled sensitivity, specificity, diagnostic odds ratio (DOR), and area under the receiver operating characteristic (ROC) curve (AUC). Quality was assessed using QUADAS-2. **Results**: Data from four studies (*n* = 441 patients) were pooled. We estimated a pooled sensitivity of 82.8% (95% CI 71.4–90.3%) and a specificity of 88.4% (95% CI 82.7–92.4%) for predicting anti-TNF non-response. The pooled AUC was 0.899 (95% CI 0.858–0.940), with low heterogeneity (I^2^ = 16.0%). The pooled DOR was 36.7 (95% CI 15.7–85.8), with a positive likelihood ratio of 7.1 and a negative likelihood ratio of 0.19. Risk of bias was low across the studies. **Conclusions**: Higher serum OSM was associated with treatment failure in IBD patients receiving anti-TNF therapy, with a pooled AUC of 0.899 in assay-stratified analysis and 0.820 when all studies were included. However, the small number of available studies, substantial variability in OSM cutoffs (14–233.6 pg/mL) and assay heterogeneity limit the robustness and generalizability and call for additional prospective data to more-robustly characterize the clinical utility of OSM.

## 1. Introduction

Tumor necrosis factor-α antagonists (anti-TNFs), including infliximab and adalimumab, are recommended for the treatment of moderate-to-severe IBD [1]. Unfortunately, up to a third of patients demonstrate primary non-response to anti-TNF-α, and up to 50% may experience secondary loss of response during maintenance therapy [2,3]. To reduce the incidence of therapeutic failures and potential toxicity from drug exposures, evidence is needed to guide the selection of patients who are likely to benefit from the TNF-α antagonist.

Although current biomarkers, including C-reactive protein, fecal calprotectin, and therapeutic drug monitoring, are useful for assessing disease severity and ongoing treatment response, they offer limited predictive value for preselecting appropriate patients for TNF-α antagonist therapy [4,5]. Limited emerging evidence suggests that higher pre-treatment levels of oncostatin M (OSM), a pleiotropic cytokine of the interleukin-6 family, generally predict non-response to TNF-α antagonist, making it a promising candidate biomarker for therapeutic selection [6,7]. OSM, produced by activated T cells, monocytes, and macrophages, is believed to trigger pro-inflammatory signaling via JAK/STAT pathways, independent of TNF-α, making it especially suited for predicting TNF-α non-response [6].

Earlier studies of OSM showed that mucosal expression strongly predicted non-response to TNF-α antagonists [6]. There is growing interest in whether serum or plasma OSM, a relatively more practical biomarker, has similar predictive value. While the majority of the evidence suggests that serum or plasma OSM has strong predictive value, the positive studies have mostly come from small observational studies [8,9,10,11]. The largest individual study to date failed to demonstrate a predictive value of serum OSM in identifying primary non-responders to TNF-α antagonists [12]. In light of this, a systematic review and meta-analysis to guide both further research and clinical decision-making is warranted. This study is an attempt to generate pooled estimates of sensitivity, specificity, and area under the curve (AUC) of OSM in predicting primary non-response in the context of IBD. Evidence from this work has the potential to guide future research and efforts towards shaping guidelines for the use of OSM in preselecting IBD patients for biologic therapy.

## 2. Methods

### 2.1. Study Reporting Guidelines

This systematic review and meta-analysis were conducted in accordance with the Preferred Reporting Items for Systematic Reviews and Meta-Analyses of Diagnostic Test Accuracy Studies (PRISMA-DTA, File S1) guidelines [13,14]. We note that the review protocol was not prospectively registered in PROSPERO or another systematic review registry. To improve transparence, the entire search criteria is included as a Supplemental File.

### 2.2. Search Strategy

A comprehensive literature search was performed in PubMed, Embase, Cochrane Library, and Web of Science from inception through March 2026 (File S2). The search strategy combined terms related to IBD, oncostatin M, anti-TNF therapy, and therapeutic response. Search terms for PubMed, Embase, and Web of Science are included as a Supplemental File. 

### 2.3. Eligibility Criteria

We included studies that (a) enrolled adult patients (≥18 years) with a confirmed diagnosis of Crohn’s disease or ulcerative colitis; (b) measured baseline serum or plasma oncostatin M concentrations prior to initiation of anti-TNF therapy (infliximab or adalimumab); (c) assessed therapeutic response to anti-TNF therapy using clinical, endoscopic, or biochemical endpoints; (d) reported sufficient data to construct 2 × 2 contingency tables or reported diagnostic measures such as sensitivity, specificity, AUC, or true positive/false positive/true negative/false negative counts; and (e) were published as full-text original research articles.

We excluded studies that (1) measured only mucosal or tissue OSM expression without serum levels; (2) were implemented in pediatric populations; (3) assessed response to non-anti-TNF biologics only (e.g., vedolizumab, ustekinumab); (4) were conference abstracts, case reports, reviews, or editorials without original data; or (5) had overlapping patient populations with other included studies. In the event that multiple studies had overlapping data or cohorts, we selected the publication with the higher sample size.

### 2.4. Study Selection

Two reviewers independently screened titles and abstracts for potential eligibility. Subsequently, full-text articles of potentially relevant studies were retrieved and assessed against the pre-specified inclusion and exclusion criteria. Disagreements were resolved by consensus or consultation with a third reviewer.

### 2.5. Data Extraction

Data were extracted independently by two reviewers using a standardized data extraction form. We extracted data on study characteristics such as first author, publication year, country, study design, enrollment period, and funding source. We also extracted patient-level data, including sample size, IBD type, age, sex distribution, disease duration, disease location and extent, disease activity scores, and history of biologic exposure. Data on the specific anti-TNF agent used (including type, dosing, and duration of use, if available) were extracted. For the purpose of this study, we restricted analyses to studies that used serum or plasma OSM. Assays were extracted as reported in the full manuscripts. Assay-related data, such as measurement method (ELISA, chemiluminescent immunoassay), assay manufacturer, and OSM threshold/cutoff value used, were extracted when available. Furthermore, we also extracted the definition of therapeutic response or non-response (including clinical remission criteria (Harvey–Bradshaw Index, partial Mayo score), endoscopic endpoints (mucosal healing, SES-CD, Mayo endoscopic subscore), and timing of outcome assessment). For diagnostic parameters, we extracted data on true positives (TPs), false positives (FPs), true negatives (TNs), false negatives (FNs), sensitivity, specificity, positive predictive value (PPV), negative predictive value (NPV), area under the receiver operating characteristic curve (AUC) with 95% confidence intervals, and optimal OSM threshold values.

### 2.6. Definition of Index Test and Reference Standard

The primary or index assay was defined as baseline serum or plasma oncostatin M concentration measured prior to or at initiation of anti-TNF therapy. We defined high OSM using study-specific thresholds. Patients were categorized as positive if OSM levels were reported as high, indicative of a high baseline risk for non-response to anti-TNF therapy.

The reference standard was therapeutic response to anti-TNF therapy, defined according to individual study criteria. Response definitions included clinical remission (Harvey–Bradshaw Index 5 for Crohn’s disease; partial Mayo score 2 for ulcerative colitis), mucosal healing (simple endoscopic score for Crohn’s disease (SES-CD) 3; Mayo endoscopic subscore ≤ 1 for ulcerative colitis) or clinical response (decrease in HBI ≥ 3 or decrease in partial Mayo score ≥ 3 from baseline). For the purposes of this meta-analysis, diagnostic accuracy was assessed in terms of predicting non-response (i.e., high OSM predicting treatment failure). Thus, we defined true positive (TP) as high OSM and non-response to anti-TNF, false positive (FP) as high OSM but response to anti-TNF, true negative (TN) as low OSM and response to anti-TNF, and false negative (FN) as low OSM but non-response to anti-TNF.

### 2.7. Quality Assessment

The methodological quality of the included studies was assessed using the Quality Assessment of Diagnostic Accuracy Studies-2 (QUADAS-2) tool [15]. This tool evaluates risk of bias and applicability concerns across four domains: patient selection, index test, reference standard, and flow and timing. Each domain was rated as low, high, or unclear risk of bias. Two reviewers independently assessed study quality, with disagreements resolved by discussion.

### 2.8. Statistical Analysis

All analyses were conducted using Stata 17 (StataCorp, College Station, TX, USA). Using the midas command in Stata, pooled estimates of sensitivity and specificity were calculated using a bivariate random-effects model [16]. Subsequently, a hierarchical summary receiver operating characteristic (HSROC) curve was constructed to visualize the trade-off between sensitivity and specificity across studies and to estimate the summary operating point. We used the “metan” command to generate pooled AUC values using a random-effects model with inverse-variance weighting.

### 2.9. Heterogeneity Assessment

The I^2^ statistic and Cochran’s Q test were used to define statistical heterogeneity. Low, moderate, and high heterogeneity were defined by I^2^ values of 25%, 50%, and 75%, respectively [17]. Due to the small number of eligible studies, subgroup analyses were deferred. Sensitivity analyses included a post hoc exclusion of Verstockt et al. [7,12]. from the pooled AUC due to the lack of data on sensitivity and specificity, which made it challenging to interpret the reported AUC. Because of the small number of available studies and sample size, additional sensitivity analyses were not feasible to do. It is worth mentioning that conventional heterogeneity metrics such as I^2^ and Cochran’s Q have recognized limitations in the context of diagnostic test accuracy meta-analyses. In particular, I^2^ does not account for threshold effects, which presents an expected source of variability in diagnostic meta-analyses. We included the HSROC curve as a framework for evaluating between-study variability in diagnostic performance as it explicitly models the correlation between sensitivity and specificity across different thresholds.

### 2.10. Publication Bias

Publication bias was assessed visually using Deeks’ funnel plot and statistically using Deeks’ asymmetry test [18]. To address the concern that the primary analysis may be influenced by the exclusion of the largest study (Verstockt et al., *n* = 186), we restructured analysis to include the presentation of pooled AUC results. The all-studies pooled AUC (including Verstockt et al.) is now presented as the primary result, with the quantitative immunoassay-restricted analysis presented as a secondary, assay-stratified analysis. This restructuring ensures that the more conservative and inclusive estimate is encountered first, while preserving the methodological rationale for examining assay-specific performance. The stratification by assay type is justified by fundamental differences in measurement methodology: the Olink proximity extension assay used by Verstockt et al. generates semi-quantitative normalized protein expression (NPX) values on a log_2_ scale, whereas the ELISA and chemiluminescent immunoassays used in the other studies produce absolute concentrations in pg/mL. Cross-platform comparisons of proteomic measurements have demonstrated that correlations between proximity extension assays and targeted immunoassays vary substantially by analyte, with approximately half the proteins showing poor correlation (r 0.5). Pooling across these fundamentally different measurement platforms without stratification would conflate assay-related variability with biological variability.

### 2.11. Software

All statistical analyses were performed using Stata-17 version 17.0 (StataCorp, College Station, TX, USA) with the midas, metandi, and metan packages. A two-sided *p*-value of 0.05 was considered statistically significant.

## 3. Results

### 3.1. Study Characteristics

**Figure 1:**
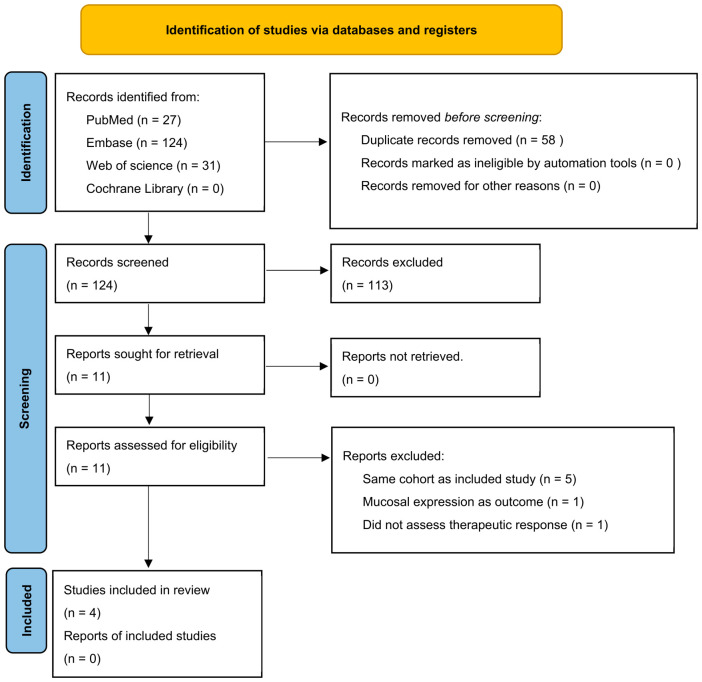
PRISMA flow diagram of literature search.

Our final analytic sample included four studies comprising 441 patients with IBD treated with anti-TNF therapy who met the inclusion criteria (Figure 1). Three studies used quantitative immunoassays (ELISA or CLIA) to measure serum/plasma OSM, while one study used a semi-quantitative proximity extension assay (Table 1). Studies were conducted in Italy, Belgium, China, and Canada, with sample sizes ranging from 40 to 186 patients. Anti-TNF agents included infliximab and adalimumab, with follow-up periods ranging from 8 to 54 weeks (Table 1).

### 3.2. Patient Characteristics

Baseline patient characteristics are presented in Table 2. Where reported, median age ranged from 36 to 43 years, and female patients comprised 39% to 54% of the study populations. Disease duration varied from 3 years (median) to 10.9 years (mean). Prior anti-TNF exposure was reported in 38% of patients in one study, while another study included only anti-TNF-naïve patients. Concomitant corticosteroid use at baseline ranged from 24% to 38%, and immunomodulator use ranged from 48% to 60%, where reported. Infliximab was the predominant anti-TNF agent, used in 46% to 100% of patients across studies (Table 2).

### 3.3. Meta-Analysis

**Figure 2:**
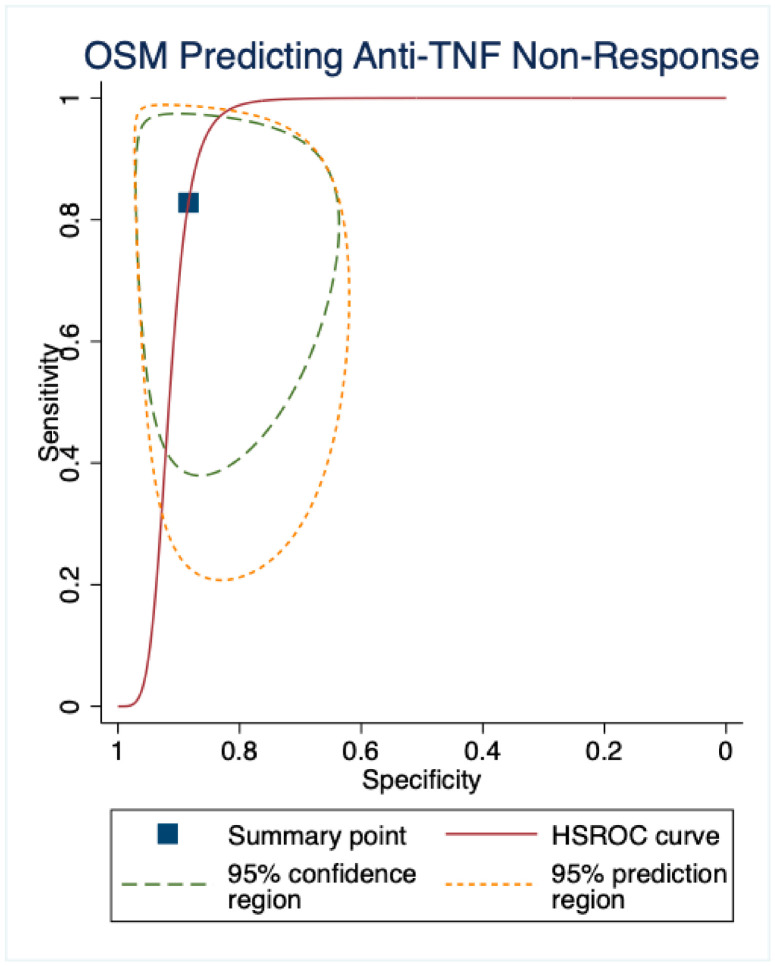
Prediction of treatment response to oncostatin M in anti-TNF therapy in IBD patients.

Sensitivity ranged from 75.0% to 90.3% and specificity from 90.7% to 95.8% (Table 3). Pooled sensitivity was 82.8% (95% CI 71.4–90.3%), and pooled specificity was 88.4% (95% CI 82.7–92.4%) (Figure 2).

### 3.4. Individual Study Results

Among the four studies using quantitative immunoassays, AUC values ranged from 0.843 to 0.940 (Table 3 and Table 4). Diagnostic odds ratios ranged from 29.4 to 99.7 (Table 4). OSM cutoff values ranged from 14 to 233.6 pg/mL (Table 3).

The study using the semi-quantitative assay (Verstockt et al.) reported an AUC of 0.522 (95% CI 0.438–0.606, *p* = 0.60), which was not significantly different from chance (Table 4).

**Figure 3:**
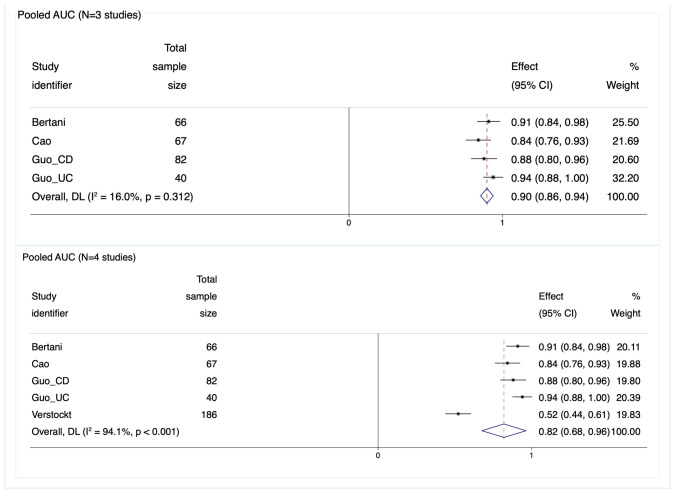
Oncostatin M for predicting response to anti-TNF: pooled estimates of area under the curve.

Overall, results were sensitive to study inclusion and assay type. When all five studies were included (*n* = 441), the pooled AUC was 0.820 (95% CI 0.676–0.963, Figure 3) with substantial heterogeneity (I^2^ = 94.1%, *p* 0.001, τ^2^ = 0.0252). In a secondary assay-stratified analysis restricted to the four studies using quantitative immunoassays (*n* = 255), the pooled AUC was 0.899 (95% CI 0.858–0.940), with low heterogeneity (I^2^ = 16.0%, *p* = 0.312, τ^2^ = 0.0003). The study using the semi-quantitative proximity extension assay (Verstockt et al.) reported an AUC of 0.522 (95% CI 0.438–0.606, *p* = 0.60), which was not significantly different from chance. Verstockt et al. did not report sensitivity, specificity, or contingency table data, precluding its inclusion in the bivariate model for pooled sensitivity and specificity. The marked difference in pooled AUC between the all-studies and assay-stratified analyses underscores the sensitivity of the findings to both study inclusion and assay methodology.

**Figure 4:**
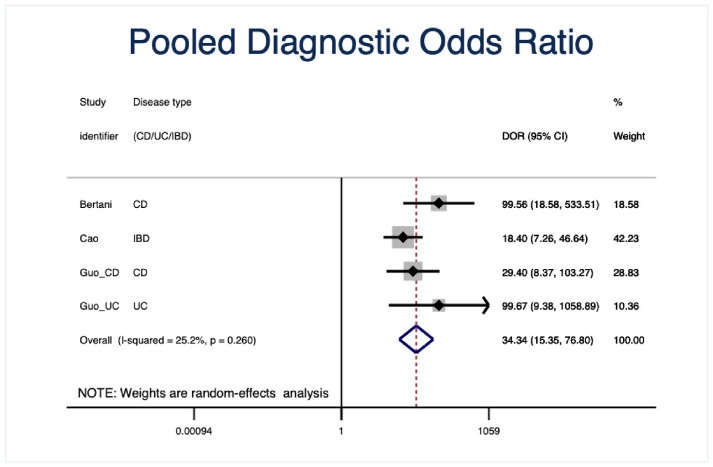
Pooled diagnostic odds ratio with use of oncostatin M for predicting treatment response in IBD patients.

The pooled diagnostic odds ratio was 36.7 (95% CI 15.7–85.8; Figure 4); the positive likelihood ratio was 7.1 (95% CI 4.6–11.1); and the negative likelihood ratio was 0.19 (95% CI 0.11–0.34) (Table 4).

### 3.5. Quality Assessment

Risk of bias was low across all domains for four studies (Table 5). One study had unclear risk of bias for patient selection and flow/timing domains (Table 5).

### 3.6. Publication Bias

The Deeks’ funnel plot asymmetry test suggested possible small-study effects (intercept = 2.92, *p* = 0.073). Given the small number of studies, the overall evidence for publication bias is weak and should be interpreted cautiously.

## 4. Discussion

This study was a systematic review and meta-analysis designed to evaluate the utility of baseline serum or plasma oncostatin M (OSM) for predicting primary non-response to or mechanistic failure of anti-TNF therapy in patients with IBD. Our primary analysis of four studies using quantitative immunoassays showed that higher pre-treatment serum OSM has good diagnostic performance for predicting anti-TNF non-response, with a pooled AUC of 0.899, sensitivity of 82.8%, and specificity of 88.4%.

Our findings are consistent with the landmark study by West et al. [6], which first established mucosal OSM as a predictor of anti-TNF non-response in IBD. Although highly specific for intestinal inflammation, mucosal sampling requires endoscopy, making it less practical. In this study, we have demonstrated that serum OSM measured by quantitative immunoassays provides a less invasive alternative with comparable predictive ability. The pooled diagnostic odds ratio of 36.7, positive likelihood ratio of 7.1, and negative likelihood ratio of 0.19 all fall within ranges considered clinically useful for diagnostic decision-making. Furthermore, the pooled estimates from the study suggest that OSM compares favorably with other emerging predictive biomarkers in IBD [7]. This includes a superior AUC relative to TREM-1(2) (AUC of 0.8) for the prediction of anti-TNF response. Similarly, in a cell-centered meta-analysis of identified baseline predictors of anti-TNF non-response in biopsy and blood samples, no single biomarker achieved the discriminative ability observed for serum OSM in our pooled analysis [19].

The ability to predict anti-TNF non-response prior to treatment initiation has significant clinical implications. Given that approximately one-third of IBD patients demonstrate primary non-response to anti-TNF therapy and up to 50% experience secondary loss of response during maintenance [2,3], a biomarker that identifies these patients could facilitate earlier transition to alternative mechanisms of action, such as vedolizumab, ustekinumab, or JAK inhibitors. This approach could reduce exposure to ineffective therapy, minimize treatment-related adverse events, and potentially improve long-term outcomes by achieving disease control more rapidly [3,20].

Several sources of clinical heterogeneity across the included studies warrant discussion. The studies differed in key demographic and clinical parameters. This included disease populations (Crohn’s disease alone vs. mixed IBD cohorts), outcome definitions (clinical remission, mucosal healing, endoscopic remission, and clinical response), timing of response assessment (ranging from 8 to 54 weeks), prior anti-TNF exposure status (anti-TNF-naïve vs. mixed populations), and assay methodologies (ELISA, chemiluminescent immunoassay, plus proximity extension assay). 

Overall, studies reporting more-stringent endpoints, such as endoscopic remission, are expected to yield higher non-response rates, thereby affecting the positive predictive value of OSM. Differences in follow-up duration across the included studies may capture different phases of treatment response, and inclusion of biologic-experienced patients may introduce confounding related to treatment-refractory disease. Furthermore, the included studies combined patients with Crohn’s disease and ulcerative colitis despite well-recognized biological and therapeutic differences among these entities. The small number of studies and the enrollment of mixed IBD populations in most of them precluded meaningful disease-specific subgroup analyses. The earliest tissue-level work by West et al. demonstrated that mucosal OSM predicted anti-TNF non-response in both Crohn’s disease and ulcerative colitis, providing a biological rationale for evaluating serum OSM across both entities; however, differential predictive performance cannot be excluded and should be examined in future studies. In addition, pooled estimates from a small number of studies may be unstable and disproportionately influenced by individual studies.

The wide range of optimal cutoff values across studies (14 to 233.6 pg/mL) [7,8,9,10,11]. reflects differences in assay platforms, calibration standards, and patient populations.

Hence, there is a need to standardize OSM assays and determine an optimal cutoff for IBD patients. The high specificity reported in the studies (90.7% to 95.8%) suggests that elevated serum OSM may be particularly useful for ruling in non-response, while the sensitivity (75.0% to 90.3%) suggests the possibility of missing non-responders and potentially inappropriately targeting IBD patients with anti-TNF agents. The lack of a validated, universally applicable cutoff value is currently the most significant barrier to translating serum OSM into routine clinical practice. Pooling sensitivity and specificity across studies with markedly different thresholds may obscure the true diagnostic performance of OSM, as each study’s operating point lies at a different position on the ROC curve. The bivariate random-effects model and HSROC framework used in our analysis specifically account for threshold variability by modeling the inherent trade-off between sensitivity and specificity. However, the approach cannot fully compensate for the clinical implications of the lack of a standardized threshold. International collaborative efforts to standardize OSM assay methodology, establish reference ranges, and determine clinically validated thresholds through large, prospective, multi-center studies are needed before any specific cutoff can be recommended for clinical use. Future studies should also report full ROC curve data to facilitate individual patient data meta-analyses and threshold optimization.

Current clinical practice relies on biomarkers such as C-reactive protein, fecal calprotectin, and therapeutic drug monitoring to assess disease activity and response [21]. However, these markers offer limited predictive value for preselecting appropriate patients for specific biologic therapies [21]. Serum OSM has the potential to fulfill this unmet need by providing pre-treatment prognostic information that could guide initial therapy selection rather than reactive treatment changes after treatment failure.

There was significant heterogeneity, largely driven by one study [12]. When this study was included in the sensitivity analysis, heterogeneity increased substantially (from 16% to 94%), while the AUC decreased to 0.820. It should be noted that conventional heterogeneity metrics such as I^2^ have recognized limitations in diagnostic test accuracy meta-analyses. Unlike intervention meta-analyses, diagnostic meta-analyses are subject to threshold effects, in which studies using different cutoff values yield correlated trade-offs between sensitivity and specificity that are not captured by I^2^. Hence, the low I^2^ of 16.0% in the assay-stratified analysis should be interpreted as reflecting limited statistical heterogeneity in the context of a small number of studies, where power to detect heterogeneity is inherently low. The HSROC curve, which accounts for the correlation between sensitivity and specificity, provides a more appropriate framework for evaluating between-study variability in this context.

The differences in results from Verstockt et al. (AUC 0.522) warrant further discussion, as this study represents the largest cohort (*n* = 186, approximately 42% of the total patient population) and the strongest contradictory evidence. Several methodological factors likely contribute to this discrepancy. First, Verstockt et al. used the Olink proximity extension assay, which generates semi-quantitative normalized protein expression (NPX) values on a log_2_ scale rather than absolute concentrations in pg/mL. Cross-platform comparisons have demonstrated that correlations between proximity extension assays and targeted quantitative immunoassays vary substantially by analyte, with approximately half of the proteins showing poor correlation. This measurement difference is not merely technical—it affects the ability to establish clinically meaningful cutoff values and may attenuate discriminative performance. Second, Verstockt et al. used the more stringent endpoint of endoscopic remission, which may capture a different aspect of treatment response than the clinical remission or mucosal healing endpoints used in other studies. Perhaps most importantly, the study did not report sensitivity, specificity, or contingency table data, making it impossible to generate pooled estimates. However, even within the Verstockt study, mucosal OSM did predict anti-TNF response (AUC 73.7%), suggesting that the negative serum finding may reflect assay-related measurement limitations rather than a true absence of biological associations. This study was included in pooled estimates of AUC, which was overall clinically significant.

This study has several strengths. First, the predictive value of OSM is biologically plausible. The available evidence suggests that OSM signals through JAK/STAT pathways independently of TNF-α, and patients with high OSM-driven inflammation may have disease that is inherently less responsive to anti-TNF therapy [6]. Additionally, this study is strengthened by a comprehensive literature search, established diagnostic validity methods, and low heterogeneity in the primary analysis (I^2^ = 16.0%). Limitations of this study include the small number of studies (*n* = 5) [8,10,11], heterogeneous outcome definitions, wide variation in OSM cutoff values (14–233.6 pg/mL), and predominantly single-center designs that require external validation [8,10,11]. Finally, we acknowledge that pooling across heterogeneous endpoints can inflate diagnostic performance. However, in this context, such inflation is unlikely given the similarities in reported sensitivity and specificity across the studies. Excluding the Verstockt study from the sensitivity analyses was necessary, as it was the only study to rely on a semi-quantitative assay. Our efforts to obtain the original data from the authors were not successful.

In conclusion, this meta-analysis demonstrates that elevated baseline serum OSM shows promise as a biomarker for predicting anti-TNF non-response in IBD, with moderate-to-high diagnostic accuracy in pooled analyses, pending additional validation studies. The results presented here are preliminary but consistent with the evolving data in this field. There is significant heterogeneity in assay methods, outcome definitions, and threshold values, which along with the limited number of studies makes the pooled estimates modest, especially when Verstockt et al. is included. Clinically, these findings are especially useful for driving hypotheses for future studies. Overall, the data presented here underscores the need for large, prospective, multi-center, and standardized validation studies to improve understanding of the potential clinical utility and diagnostic value of OSM in prediciting response to anti-TNF.

Registration/protocol: The review was not registered. The protocol is included as part of the manuscript.

## Figures and Tables

**Table 1:** 0: Characteristics of studies included in meta-analysis.

Study	Year	Country	Study Design	Disease	Sample Size	Anti-TNF Agent(s)	Outcome Definition	Follow-Up	OSM Sample	Assay Method	OSM Cutoff (pg/mL)
Bertani et al. [8]	2022	Italy	Prospective cohort	CD	66	IFX, ADA	Mucosal healing (SES-CD ≤ 3)	54 weeks	Serum	ELISA	14
Cao et al. [10]	2022	China	Prospective cohort	CD + UC	67	IFX	Clinical response (HBI or pMS ≤ 3)	Maintenance phase	Serum	CLIA	83
Guo et al. [11]	2022	Canada	Retrospective cohort	CD	82	IFX, ADA	Clinical remission (HBI ≤ 5)	52 weeks	Plasma	ELISA	168.7
Guo et al. [11]	2022	Canada	Retrospective cohort	UC	40	IFX, ADA	Clinical remission (pMS ≤ 2)	52 weeks	Plasma	ELISA	233.6
Verstockt et al. † [12]	2021	Belgium	Prospective cohort	CD + UC	186	IFX, ADA	Endoscopic remission ‡	6 months (CD)/8–14 weeks (UC)	Serum	Olink PEA	NE

† Serum OSM was not predictive of anti-TNF response (*p* = 0.60); no cutoff established. ‡ Endoscopic remission defined as complete absence of ulcerations (CD) or Mayo endoscopic subscore 0–1 (UC).

**Table 2:** Baseline patient characteristics from studies included in meta-analysis.

Characteristic	Bertani 2022 [8] (*n* = 66)	Cao 2022 [10] (*n* = 67)	Guo 2022 CD [11] (*n* = 82)	Guo 2022 UC [11] (*n* = 40)	Verstockt 2021 [12] (*n* = 186) †
Mean/median age (range/IQR)	36 (25–48) *	NR	43.0 (18–78)	39.4 (21–73)	NR
Female (%)	26 (39)	NR	44 (54)	19 (48)	NR
Disease type					
Crohn’s disease (%)	66 (100)	50 (75)	82 (100)	-	NR
Ulcerative colitis (%)	0 (0)	17 (25)	-	40 (100)	NR
Disease duration, years	3 (median)	NR	10.9 ± 11.3	7.6 ± 8.8	NR
Prior anti-TNF use	25 (38)	NR	NR	0 (0) *	NR
Prior IBD surgery	NR	NR	27 (33)	0 (0)	NR
Baseline disease severity					
SES-CD, median (IQR)	8 (7–12)	NR	NR	-	NR
HBI, median (IQR)	NR	NR	7 (2)	-	NR
pMS, median (IQR)	-	NR	-	8 (1)	NR
Concomitant medications					
Steroids (%)	25 (38)	NR	20 (24)	11 (28)	NR
Immunomodulators (%)	Excluded	NR	49 (60)	19 (48) *	NR
Anti-TNF received (%)					
Infliximab	45 (68)	67 (100)	38 (46)	28 (70)	NR
Adalimumab	21 (32)	0 (0)	44 (54)	12 (30)	NR

Guo et al. [11] reported combination therapy with azathioprine or methotrexate. † Verstockt et al. [12] reported 91 patients achieved remission and 95 did not; detailed baseline characteristics for the anti-TNF cohort were not reported separately. * Median age and IQR (interquartile range). NR = not reported.

**Table 3:** Summary of contingency data and pooled estimates of sensitivity and specificity.

Study	Disease	N	TP	FP	FN	TN	Cutoff (pg/mL)	Sensitivity, % (95% CI)	Specificity, % (95% CI)	PPV, % (95% CI)	NPV, % (95% CI)
Bertani (2022) [8]	CD	66	28	3	3	32	14	90.3 (79.5–100.0)	91.4 (81.8–100.0)	90.3 (79.5–100.0)	91.4 (81.8–100.0)
Cao (2022) [10]	IBD	67	19	4	4	40	83	82.6 (67.1–98.1)	90.9 (82.4–99.4)	82.6 (67.1–98.1)	90.9 (82.4–99.4)
Guo_CD (2022) [11]	CD	82	21	5	7	49	168.7	75.0 (58.5–91.5)	90.7 (82.6–98.9)	80.8 (65.6–95.9)	87.5 (78.9–96.1)
Guo_UC (2022) [11]	UC	40	13	1	3	23	233.6	81.3 (62.1–100.0)	95.8 (87.8–100.0)	92.9 (79.4–100.0)	88.5 (75.4–100.0)
Verstockt (2021)‚ Ä † [12]	IBD	186	N/A	N/A	N/A	N/A	NE	N/A	N/A	N/A	N/A
Pooled	IBD	255	81	13	17	144	-	82.8 (71.4–90.3)	88.4 (82.7–92.4)	-	-

Abbreviations: CD = Crohn’s disease; UC = ulcerative colitis; IBD = inflammatory bowel disease; TP = true positive; FP = false positive; FN = false negative; TN = true negative; NE = not established; PPV = positive predictive value; NPV = negative predictive value. † Serum OSM did not discriminate responders from non-responders (*p* = 0.60) and was not predictive of anti-TNF response; no cutoff established; contingency data not calculable; diagnostic performance metrics not applicable.

**Table 4:** Pooled estimates of areas under the curve and likelihood ratios.

Study	AUC (95% CI)	DOR (95% CI)	LR+	LR−
Bertani (2022) [8]	0.910 (0.840–0.990)	99.6 (22.0–449.5)	10.5	0.11
Cao (2022) [10]	0.898 (0.800–0.959)	47.5 (11.5–196.2)	9.1	0.19
Guo_CD (2022) [11]	0.880 (0.790–0.960)	29.4 (8.9–97.2)	8.1	0.28
Guo_UC (2022) [11]	0.938 (0.870–1.000)	99.7 (10.0–990.5)	19.5	0.20
Verstockt (2021) † [12]	0.522 (0.438–0.606)	N/A	N/A	N/A
Pooled (all)	0.820 (0.676–0.963)	N/A	N/A	N/A
Pooled (quantitative assays)	0.820 (0.676–0.963)	N/A	N/A	N/A

† Serum OSM AUC was not significantly different from chance (*p* = 0.60); DOR and likelihood ratios not calculable. AUC = area under the curve; DOR = diagnostic odds ratio; LR+ = positive likelihood ratio; LR− = negative likelihood ratio.

**Table 5:** Quality assessment of studies included in meta-analysis.

Domain	Bertani 2022 [8]	Cao 2022 [10]	Guo 2022 [11]	Verstockt 2021 [12]
Risk of bias				
Patient selection	Low	Unclear	Low	Low
Index test	Low	Low	Low	Low
Reference standard	Low	Low	Low	Low
Flow and timing	Low	Unclear	Low	Low
Applicability concerns				
Patient selection	Low	Low	Low	Low
Index test	Low	Low	Low	Low
Reference standard	Low	Low	Low	Low

## Data Availability

The original contributions presented in this study are included in the article/Supplementary Material. Further inquiries can be directed to the corresponding author.

## Supplementary Materials

The following supporting information can be downloaded at: https://www.mdpi.com/article/10.3390/clinpract16070124/s1, File S1: PRISMA 2020 Checklist; File S2: Search terms.
