# Supplementary material for: Serum or Plasma Oncostatin M for Predicting Primary Non-Response to Tumor Necrosis Factor-α Antagonist Therapy in Inflammatory Bowel Disease: A Systematic Review and Meta-Analysis
Source: Clin Pract. 2026 Jul 2;16(7):124. doi: 10.3390/clinpract16070124 (PMC13408984; doi:10.3390/clinpract16070124)
Supplement: Supplementary file 1 [file clinpract-16-00124-s001.zip › File S2 Search terms.pdf]

Search terms:

PUBMED:
("Inflammatory Bowel Diseases"[Mesh] OR "Crohn Disease"[Mesh] OR "Colitis, Ulcerative"[Mesh]
OR "inflammatory bowel disease"[tiab] OR IBD[tiab]
OR "crohn disease"[tiab] OR "ulcerative colitis"[tiab])AND("Oncostatin M"[Mesh] OR "oncostatin m"[tiab] OR "oncostatin-m"[tiab] OR OSM[tiab]) AND("Treatment Outcome"[Mesh] OR "Tumor Necrosis Factor-alpha"[Mesh] OR "Antibodies, Monoclonal"[Mesh]
OR "anti tnf"[tiab] OR infliximab[tiab] OR adalimumab[tiab]
OR golimumab[tiab] OR certolizumab[tiab] OR biologic*[tiab]
OR "treatment response"[tiab] OR remission[tiab]
OR "mucosal healing"[tiab] OR nonrespons*[tiab])
AND (predict*[tiab] OR prognos*[tiab] OR "predictive value"[tiab]
OR sensitivity[tiab] OR specificity[tiab]
OR "receiver operating characteristic"[tiab] OR ROC[tiab] OR AUC[tiab]) AND humans[MeSH Terms]
AND
english[lang]

EMBASE:
('inflammatory bowel disease'/exp OR 'Crohn disease'/exp OR 'ulcerative colitis'/exp
OR 'inflammatory bowel disease':ti,ab,kw OR ibd:ti,ab,kw
OR 'crohn disease':ti,ab,kw OR 'ulcerative colitis':ti,ab,kw)
AND
('oncostatin M'/exp OR 'oncostatin m':ti,ab,kw OR 'oncostatin-m':ti,ab,kw OR osm:ti,ab,kw
OR 'biological marker'/exp OR biomarker*:ti,ab,kw OR 'predictive marker':ti,ab,kw)
AND
('treatment response'/exp OR 'tumor necrosis factor alpha'/exp OR 'monoclonal antibody'/exp
OR 'anti tnf':ti,ab,kw OR infliximab:ti,ab,kw OR adalimumab:ti,ab,kw
OR golimumab:ti,ab,kw OR certolizumab:ti,ab,kw OR biologic*:ti,ab,kw
OR 'treatment response':ti,ab,kw OR remission:ti,ab,kw
OR 'mucosal healing':ti,ab,kw OR nonrespons*:ti,ab,kw)
AND
(predict*:ti,ab,kw OR prognos*:ti,ab,kw OR 'predictive value':ti,ab,kw
OR sensitivity:ti,ab,kw OR specificity:ti,ab,kw
OR 'receiver operating characteristic':ti,ab,kw OR roc:ti,ab,kw OR auc:ti,ab,kw)
AND [humans]/lim AND [english]/lim

Title or abstract:
('inflammatory bowel disease'/exp OR 'Crohn disease'/exp OR 'ulcerative colitis'/exp
OR 'inflammatory bowel disease':ti,ab OR ibd:ti,ab
OR 'crohn disease':ti,ab OR 'ulcerative colitis':ti,ab)
AND
('oncostatin M'/exp OR 'oncostatin m':ti,ab OR 'oncostatin-m':ti,ab OR osm:ti,ab)
AND
('treatment response'/exp OR 'tumor necrosis factor alpha'/exp OR 'monoclonal antibody'/exp
OR 'anti tnf':ti,ab OR infliximab:ti,ab OR adalimumab:ti,ab
OR golimumab:ti,ab OR certolizumab:ti,ab OR biologic*:ti,ab
OR 'treatment response':ti,ab OR remission:ti,ab
OR 'mucosal healing':ti,ab OR nonrespons*:ti,ab)
AND
(predict*:ti,ab OR prognos*:ti,ab OR 'predictive value':ti,ab
OR sensitivity:ti,ab OR specificity:ti,ab
OR 'receiver operating characteristic':ti,ab OR roc:ti,ab OR auc:ti,ab)

AND
[humans]/lim AND [english]/lim

WEB of science:
#1 'inflammatory bowel disease'/exp OR 'Crohn disease'/exp OR 'ulcerative colitis'/exp OR 'inflammatory bowel disease<em>':ti,ab,kw OR ibd:ti,ab,kw OR 'crohn disease':ti,ab,kw OR 'ulcerative colitis':ti,ab,kw

#2 'oncostatin M'/exp OR 'oncostatin m':ti,ab,kw OR 'oncostatin-m':ti,ab,kw OR osm:ti,ab,kw OR 'biological marker'/exp OR biomarker<em>:ti,ab,kw OR 'predictive marker':ti,ab,kw

#3 'treatment response'/exp OR 'tumor necrosis factor alpha'/exp OR 'monoclonal antibody'/exp OR 'anti tnf':ti,ab,kw OR infliximab:ti,ab,kw OR adalimumab:ti,ab,kw OR golimumab:ti,ab,kw OR certolizumab:ti,ab,kw OR biologic<em>:ti,ab,kw OR 'treatment response':ti,ab,kw OR remission:ti,ab,kw OR 'mucosal healing':ti,ab,kw OR nonrespons:ti,ab,kw

#4 predict<em>:ti,ab,kw OR prognos:ti,ab,kw OR 'predictive value':ti,ab,kw OR sensitivity:ti,ab,kw OR specificity:ti,ab,kw OR 'receiver operating characteristic':ti,ab,kw OR roc:ti,ab,kw OR auc:ti,ab,kw

#5 #1 AND #2 AND #3 AND #4

#6 Limits: [humans]/lim AND [english]/lim
